# Microdose lithium reduces cellular senescence in human astrocytes - a potential pharmacotherapy for COVID-19?

**DOI:** 10.18632/aging.103449

**Published:** 2020-06-13

**Authors:** Tania Viel, Shankar Chinta, Anand Rane, Manish Chamoli, Hudson Buck, Julie Andersen

**Affiliations:** 1 Laboratory of Neuropharmacology of Aging, School of Arts, Sciences and Humanities, Universidade de São Paulo, Sao Paulo, Brazil; 2 Touro University California, Vallejo, CA 94592, USA; 3 Buck Institute for Research on Aging, Novato, CA 94945, USA; 4 Department of Physiological Sciences, Santa Casa de Sao Paulo School of Medical Sciences, Sao Paulo, Brazil

**Keywords:** senolytics, lithium, COVID-19, cell senescence, chronic inflammation

## Abstract

Cell senescence is a process that causes growth arrest and the release of a senescence associated secretory phenotype (SASP), characterized by secretion of chemokines, cytokines, cell growth factors and metalloproteases, leading to a tissue condition that may precipitate cancers and neurodegenerative processes. With the recent pandemic of coronavirus, senolytic drugs are being considered as possible therapeutic tools to reduce the virulence of SARS-CoV-2. In the last few years, our research group showed that lithium carbonate at microdose levels was able to stabilize memory and change neuropathological characteristics of Alzheimer’s disease (AD). In the present work, we present evidence that low-dose lithium can reduce the SASP of human iPSCs-derived astrocytes following acute treatment, suggesting that microdose lithium could protect cells from senescence and development of aging-related conditions. With the present findings, a perspective of the potential use of low-dose lithium in old patients from the “high risk group” for COVID-19 (with hypertension, diabetes and chronic obstructive pulmonary disease) is presented.

## INTRODUCTION

Since the beginning of the coronaviral burst in December 2019, SARS-CoV-2 has widely spread in more than 50 countries around the world. The increased fatality of COVID-19 amongst older versus younger individuals has become more evident [1].

The aging process is characterized by increased levels of oxidative stress and chronic inflammation contributing to many age-related pathologies [2, 3]. It has been suggested that increases in inflammation may be promoted by the release of pro-inflammatory and other factors from senescent cells as part of what is known as the senescence-associated secreted phenotypes (SASP) [4, 5], which includes increase in senescence-associated β-galactosidase (SA-β-gal) activity, increased levels of the cyclin-dependent kinase (CDK) inhibitors p16 and p21, and pro-inflammatory cytokines including IL-6, IL-8 and IL-1α [6]. The SASP promotes the development of an inflammatory environment leading to tissue frailty contributing to many diseases including cancer, chronic obstructive pulmonary disease, diabetes and neurodegenerative diseases [5, 7–9].

In a recent research perspective, the use of senolytic drugs was suggested for the treatment and prevention of COVID-19 [10]. These drugs induce the apoptosis of senescent cells and reduce production of the SASP, reducing vulnerability to chronic diseases [11]. The authors described how many FDA-approved drugs including azithromycin, doxycycline and chloroquine have been shown to act as senolytics.

Recently our research group and others have identified additional compounds that may also inhibit the inflammation associated with aging and neurodegenerative diseases. Lithium in microdose [12, 13], for example, was shown to enhance the maintenance of memory, decrease the density of senile plaques, and reduce neuronal cell loss both clinically and pre-clinically. A recent review highlighted the potential use of lithium as candidate for therapy of COVID-19 along with chloroquine or other drugs [14]. It is possible that one of the mechanisms by which microdose lithium may be eliciting its protective effects is via preventing inflammatory SASP induction.

**Figure 1:**
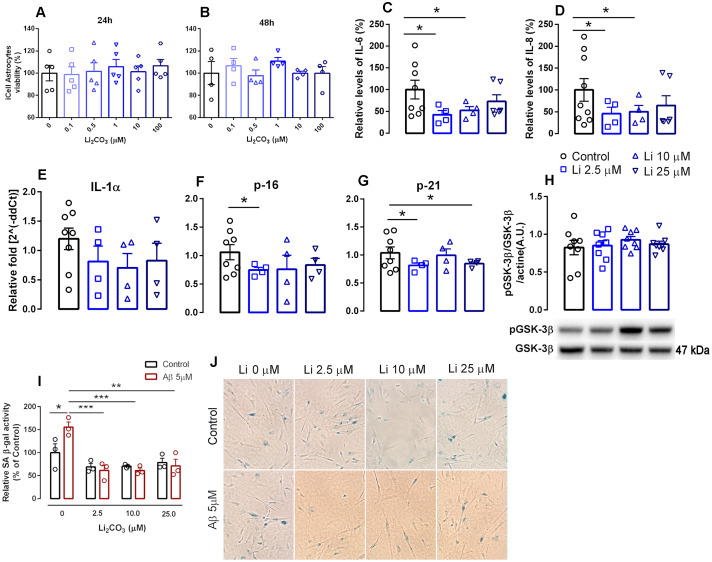
**Effects of increasing lithium concentrations on cell viability and induction of senescence and the SASP in human iPSC-derived astrocytes.** (**A**, **B**) cell viability measured by the MTT assay. Data are expressed as individual points, mean and SEM; performed in triplicate. (**C**, **D**) Relative levels of secreted IL-6 and IL-8. Conditioned media was collected 24h following induction of senescence with 1% FBS and data was normalized to cell number. (**E**–**G**) RNA isolated from human iPSCs-derived astrocytes was analyzed for IL-1α, p16^INK4a^ and p21 mRNA levels by qPCR. Transcripts were normalized to actin and are shown as fold change over control levels. (**H**) GSK-3β activation measured as the proportion of phosphorylated and total GSK-3β. Data are expressed as individual points, mean and SEM. (**I**) SA β-gal in iPSC-derived astrocytes in the absence and presence of Aβ with increasing concentrations of lithium. Values show relative amounts of SA β-gal positive cells in three independent experiments. (**J**) Representative panels of SA-β gal staining under various treatment conditions.*p<0.05; **: p<0.01; ***:p < 0.001. For (**C**–**H**), data are expressed as individual points, mean and SEM of 4-5 independent experiments.

In order to test this, human iPSCs-derived astrocytes were seeded in cell culture plates pre-coated with matrigel (Corning Matrigel Matrix, Tewksbury, MA, USA) and treated with different concentrations of Li_2_CO_3_ for 24 h and 48h. Concentrations up to 100 μM showed no toxicity in the astrocytes as determined by the MTT assay (Figure 1A, 1B). Based on this analysis and our previous pre-clinical studies [13], three concentrations (2.5 μM, 10 μM and 25 μM) were selected for subsequent experiments; treatments were maintained for 24 h.

Concentrations of the hallmark SASP factors such as IL-6 and IL-8 were measured in the conditioned culture media using ELISA kits. Treatment with 2.5 μM and 10 μM Li_2_CO_3_ promoted a 57.6% (P<0.05) and 47.5% decrease (P<0.05), respectively, in the release of IL-6 and a 54.2% (P<0.05) and 49.6% (P<0.05) decrease in the release of IL-8 compared to untreated controls. Incubation of 25 μM Li_2_CO_3_ however did not alter the release of either cytokine (Figure 1C, 1D). These data are in agreement with recent studies from our lab and others showing anti-inflammatory properties of low-dose lithium as evidenced by reductions in pro-inflammatory cytokine density [15], Toricelli et al. (Toricelli M, Evangelista SR, Buck HS, Viel TA. Microdose lithium treatment reduced inflammatory factors and neuro-degeneration in organotypic hippocampal culture of old SAMP-8 mice. Submitted to Cellular and Molecular Neurobiology, March 2020). These results are of particular interest as a very recent report shows strong association of elevated IL-6 levels with respiratory failure in COVID-19 infected patients [16].

Similar expression profiles for the senescence markers p16 and p21 and the SASP factor IL-1α were also observed following treatment with Li_2_CO_3_ compared with untreated controls. 2.5 μM Li_2_CO_3_ significantly reduced expression of p16 and p21 and 25 μM Li_2_CO_3_ also reduced p21 expression. For IL-1α, however, the decrease in expression with Li_2_CO_3_ did not reach statistical significance (Figure 1E–1G).

Interestingly, positive effects of acute treatment with low dose lithium seems not to act via known mechanism of lithium (inhibition of GSK-3β activation) [17], as no differences in phosphorylation of Ser9-GSK-3β were observed following acute treatment with low concentrations of lithium (Figure 1H). In a previous study, treatment of WI-38 fibroblasts with 20 mM lithium chloride reduced GSK3-dependent increases in p53 and p21 nuclear levels [18], indicating that microdose lithium used in the present work has different cell effects than lithium in higher concentrations.

We further confirmed the antisenescence properties of lithium using an established amyloid β-induced senescence model [19]. We observed that low dose of Li_2_CO_3_ including 2.5 μM, 10 μM and 25 μM significantly suppressed amyloid-β (Aβ) increased SA β-gal staining in astrocytes, a hallmark of cellular senescence (Figure 1I, 1J). Overall our results highlight the potential of microdose lithium (a safe FDA approved drug) in suppressing cellular senescence.

Lithium carbonate is still widely used as a therapeutic for bipolar depression [20]. Recently, low-dose lithium has begun to be considered as a disease-modifying strategy for some neurodegenerative diseases [13, 15, 21–24]. Its neuroprotective effects in pre-clinical models may be due to its anti-inflammatory properties [15, 25] Toricelli et al.^1^.

This work was originally initiated by the authors to explore the beneficial effects of low-dose lithium in brain aging and age-related neurodegenerative diseases. However, in face of the recent COVID-19 pandemic and the urgency to identify anti-viral drugs, including the potential use of FDA-approved drugs displaying senolytic properties, we believe that these findings will be important to broaden the research community therapy possibilities. The fact that microdose lithium suppresses IL-6 and recent finding correlating IL-6 level with severity of the diseases in COVID-19 patients provides a strong rationale for why lithium treatment should be tested as treatment. In this way, low-dose lithium may constitute a novel potential therapeutic to reduce the virulence of SARS-CoV-2. It is important to highlight that no side effects were verified in old people with the use of low-dose lithium [12, 26].

## MATERIALS AND METHODS

### Culture of human iPSCs-derived astrocytes

Commercially available human iPSC-derived astrocytes (iCell, # 01434) were used for our studies. Cells were seeded at 1 x 10^4^ cells/cm^2^ in cell culture plates pre-coated with matrigel (Corning Matrigel Matrix, Tewksbury, MA, USA) and cultured to 70-80% confluence. Cells were then cultured at 37°C and 5% CO_2_ in complete DMEM media (supplemented with N2 supplement and 2% penicillin/streptomycin) containing 10% fetal bovine serum (FBS). Cells were grown in physiological (3%) oxygen concentrations as previously described [27, 28]. Cells were incubated with concentrations of up to 1 mM Li_2_CO_3_ for 24-48 hrs and toxicity verified by the MTT assay.

### Determination of IL-6 and IL-8 levels

Following treatment with 2.5 μM, 10 μM and 25 μM Li_2_CO_3_ for 24h, culture medium was prepared by washing cells once in PBS followed by incubation in DMEM with 1% FBS for 24 hr. The medium was collected and stored at -80 °C. Cell numbers were determined with an automated cell counter (Thermo Scientific). ELISA assays were performed using an alphaLISA IL-6 or IL-8 Immunoassay Research Kit (Perkin Elmer) following the manufacturer’s instructions. Data was normalized to cell number and expressed as picograms per 1,000 cells.

### RT-qPCR 

Total RNA was prepared from human astrocytes using a Direct-zol RNA MiniPrep Kit (Genesee Scientific). Integrity of RNA was verified using a nanodrop system. RT-qPCR was performed using the Universal Probe Library System (Roche, South San Francisco, CA) with the following primers and probes:

IL-1a: forward (FW) 5′-ggttgagtttaagccaatcca-3′; reverse (RV) 5′-tgctgacctaggcttgatga-3′

p16^INK4a^: FW 5′-cggaaggtccctcagacatc-3′; RV 5′-aaactacgaaagcggggtgg-3′

p21: FW 5′-ccagcatgacagatttctaccac-3′; RV 5′- cttcctgtgggcggattagg-3′

actin: 5′-ACCGAGCGCGGCTACAG-3′; 5′-CTTAATGTCACGCACGATTTCC-3′

### Determination of GSK-3β activation

For protein extractions, astrocytes were collected and homogenized in lysis buffer containing 50 mM Tris pH 8.0, 150 mM NaCl, 1% NP-40, a protease inhibitor cocktail (Roche) and a phosphatase inhibitor cocktail (Sigma-Aldrich). Lysates were centrifuged at 10,000g for 10 min at 4 °C and supernatants collected. Total protein concentration was determined using the Bradford assay [29]. Proteins (10 μg) were separated by 10% sodium dodecyl sulfate-polyacrylamide gel electrophoresis (SDS-PAGE) and transferred onto PVDF membranes. Membranes were blocked with TBST containing 5% non-fat milk for 1 hour and then incubated with the primary antibody GSK-3β (Cell Signaling Technology, 9315, 1:1000) and phospho-GSK-3β (Cell Signaling Technology, 5558, 1:1000). Bands were detected using an ECL system (EMD Millipore) and quantified densitometrically. Actin (1:2000) was used as a loading control.

### Senescence-associated-β-galactosidase (SA-β-gal) assay

SA-β-gal staining was performed according to the method described by Bhat and co-workers [19]. Cells were plated at 1 x 10^4^ cells/cm^2^ in chamber slides and treated or not with 5 μM amyloid-β for 2 h. The medium was then replaced with fresh medium containing 0 μM, 2.5 μM, 10 μM or 25 μM Li_2_CO_3_. This treatment was maintained for three days after which cells were assessed for SA-β-gal activity. Positive (blue) cells were expressed as a percentage of total cell number.

### Statistical analysis

Data were expressed as means ± SEM and analyzed with the Graph Pad Prism program (GraphPad Software, San Diego, CA, version 6). Data were analyzed using one-way analysis of variance (ANOVA) followed by Bonferroni’s test. In all analyses, only probability values (*P*) less than 0.05 were considered statistically significant.

### Data availability statement

All data generated or analyzed during this study are included in this published article.
